# Single Neuron Coding of Identity in the Human Hippocampal Formation

**DOI:** 10.1016/j.cub.2020.01.035

**Published:** 2020-03-23

**Authors:** Hernan G. Rey, Belen Gori, Fernando J. Chaure, Santiago Collavini, Alejandro O. Blenkmann, Pablo Seoane, Eduardo Seoane, Silvia Kochen, Rodrigo Quian Quiroga

**Affiliations:** 1 Centre for Systems Neuroscience, University of Leicester, 15 Lancaster Rd, Leicester LE1 7HA, UK; 2 Neurosciences and Complex Systems Unit (EnyS), CONICET, Hospital El Cruce “Nestor Kirchner”, Universidad National Arturo Jauretche (UNAJ), Av. Calchaquí 5401, Buenos Aires 1888, Argentina; 3 Institute of Biomedical Engineering, University of Buenos Aires, Paseo Colon 850, Buenos Aires 1063, Argentina; 4 Institute of Electronics, Control and Signal Processing (LEICI), University of La Plata, Calle 116 s/n, La Plata B1900, Argentina

**Keywords:** single neurons, hippocampus, medial temporal lobe, memory, invariance

## Abstract

Experimental findings show the ubiquitous presence of graded responses and tuning curves in the neocortex, particularly in visual areas [1, 2, 3, 4, 5, 6, 7, 8, 9, 10, 11, 12, 13, 14, 15]. Among these, inferotemporal-cortex (IT) neurons respond to complex visual stimuli, but differences in the neurons’ responses can be used to distinguish the stimuli eliciting the responses [8, 9, 16, 17, 18]. The IT projects directly to the medial temporal lobe (MTL) [19], where neurons respond selectively to different pictures of specific persons and even to their written and spoken names [20, 21, 22]. However, it is not clear whether this is done through a graded coding, as in the neocortex, or a truly invariant code, in which the response-eliciting stimuli cannot be distinguished from each other. To address this issue, we recorded single neurons during the repeated presentation of different stimuli (pictures and written and spoken names) corresponding to the same persons. Using statistical tests and a decoding approach, we found that only in a minority of cases can the different pictures of a given person be distinguished from the neurons’ responses and that in a larger proportion of cases, the responses to the pictures were different to the ones to the written and spoken names. We argue that MTL neurons tend to lack a representation of sensory features (particularly within a sensory modality), which can be advantageous for the memory function attributed to this area [23, 24, 25], and that a full representation of memories is given by a combination of mostly invariant coding in the MTL with a representation of sensory features in the neocortex.

## Highlights

• Most human MTL neurons show a nearly binary coding of identity
• Pictures of the same person could not be distinguished from the neural responses
• The MTL binary coding contrasts with the graded coding typically seen in neocortex
• Binary coding offers advantages for the encoding and retrieval of episodic memories

## Teaser

Rey, Gori et al. show that pictures of the same identity were indistinguishable from each other from the firing of human MTL neurons. These neurons show a binary, invariant code of identity—in contrast to the graded coding typically found in the cortex—which is critical for the encoding and retrieval of episodic memories.

## Results

### Experimental Paradigm and Neural Recordings

We recorded single neuron activity during 16 sessions in six patients with drug-resistant epilepsy, who were candidates for surgical treatment and implanted with intracranial electrodes (STAR Methods). For each identity used in an experimental session, a total of five stimuli were presented: three different pictures, the name written on the screen, and the name spoken by a computer-synthesized voice. Each stimulus was presented 30 times in pseudorandom order (in contrast to the limited six trials used in many previous works). From the 450 units recorded in these sessions, the response criterion (STAR Methods; Figure S1) led to a set of 159 responses in 35 units (16 from amygdala and 19 from hippocampus).

### Invariant Responses in the Human MTL

**Figure 1:**
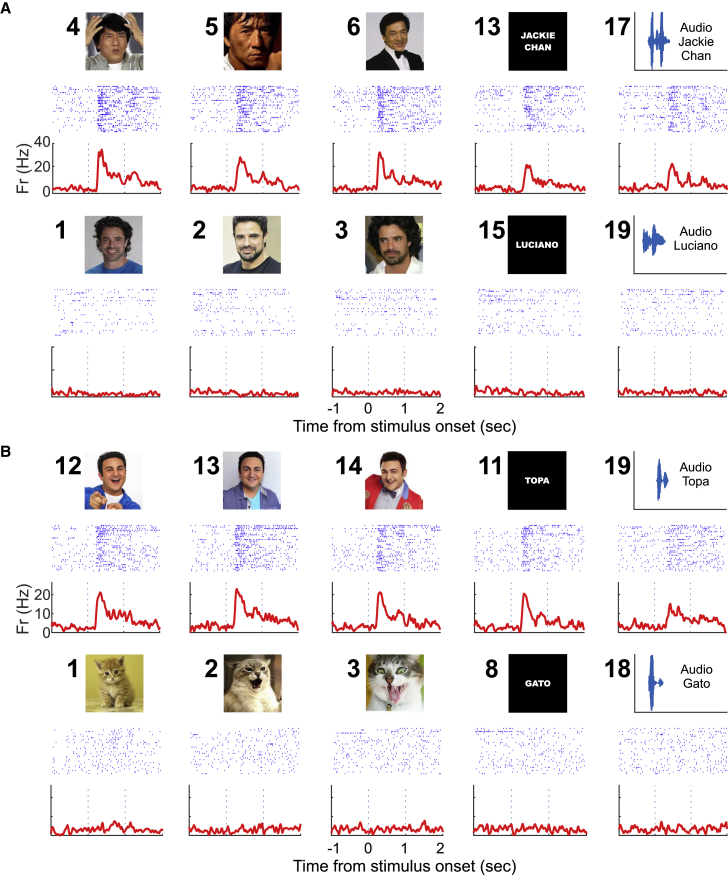
Exemplary Invariant Units (A) Responses of a unit in the left hippocampus. For each stimulus, the raster plot (blue lines represent the appearance of a spike and each row is associated to a trial; first trial is at the top and time zero is the stimulus onset) and instantaneous firing rate are shown. Stimulus numbers appear next to the stimulus pictures. The unit responded to actor “Jackie Chan” but not to another actor, “Luciano Castro.” (B) Responses of a unit in the left hippocampus. The unit responded to all stimuli associated to a TV host (“Topa”) but not to pictures of a cat or the written or spoken name “Gato” (cat). For space reasons, only 10 of 20 stimuli are shown on each panel, but there were no significant responses to the other stimuli not shown. See Figure S1 for further examples of response-eliciting stimuli and Figure S2 for another example of a neuron responding to multiple identities.

Figure 1A shows an example of a unit from the hippocampus responding to all the stimuli associated to the actor “Jackie Chan.” From an average baseline firing of 3.65 Hz (SD 3.89), upon presentation of these stimuli, the neuron increased its firing to an average of 14.19 Hz (SD 5.66,) with peaks of up to 30 Hz, in the response window. Figure 1B shows another example of a unit responding to all the stimuli associated to a TV host in Argentina. In this case, from an average baseline firing of 3.05 Hz (SD 2.76), the neuron had an average firing of 11.67 Hz (SD 4.96) in response to these stimuli. Although most units responded selectively to a single identity, there were several neurons (7 out of 35) showing responses to more than one identity. One example is shown in Figure S2, where a hippocampal neuron responded to the stimuli associated to three TV celebrities.

**Figure 2:**
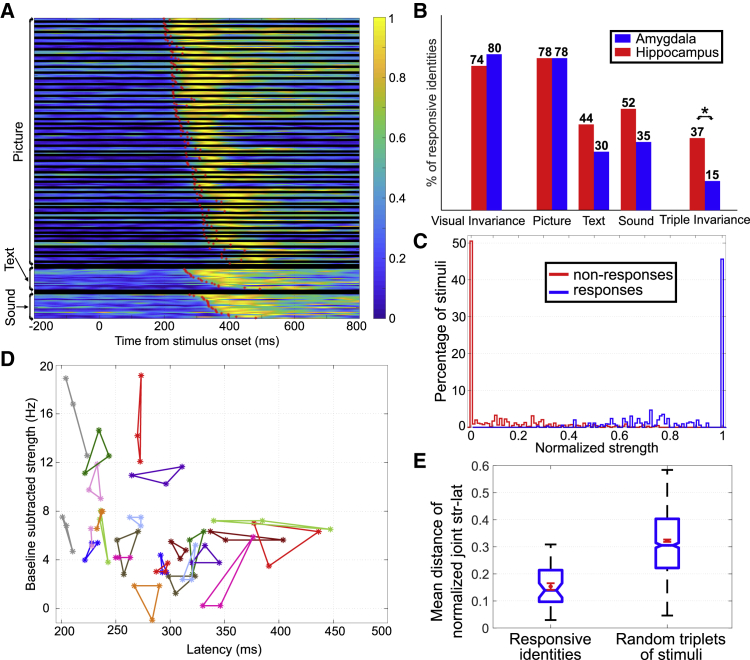
Response Characteristics of the Population of Recorded Cells (A) Normalized firing rate for all response-eliciting stimuli, separated by stimulus type (picture, text, and sound). Within the pictures, the 47 response-eliciting identities are separated by black lines and sorted by their shortest latency. Response latency for each stimulus is denoted by a red star. Figure S2 shows a neuron with 15 response-eliciting stimuli from three different identities. (B) The proportion of response-eliciting identities was evaluated in different conditions and segregated between the units in the hippocampus and amygdala. In both regions, similar proportions were found for visual stimuli (for visual invariance and for the proportion of identities showing responses to more than one picture), but larger proportions in the hippocampus were seen for text and sound stimuli. In the case of triple invariance, i.e., identities showing visual invariance and responses to the text and sound stimuli, the proportion of identities in the hippocampus was significantly larger than in amygdala (Z test, p < 0.05). (C) Histograms for the normalized strength of activity in all the responsive units, computed for all the stimuli presented in each session and separated according to whether or not they were responsive. (D) Strength (baseline corrected) and latency for each picture in 25 out of the 47 response-eliciting identities (the remaining 22 are presented in Figures S3A and S3B). Each triplet associated to a given identity is depicted as a triangle of a given color with each picture corresponding to the vertices of the triangle. (E) Average joint distance (in the strength-latency space presented in Figure 3D) for the three pictures of a given identity (left), and triplets created by randomly selecting three pictures out of the 141 pictures (right). Mean ± standard error of the mean is shown in red, while boxplots are in blue (center line, median; box limits, upper and lower quartiles; notch limits, (1.57 × interquartile range)/sqrt(n)) with the whiskers in black extending to the most extreme data points not considered as outliers. There were significant differences between the two populations (rank-sum test, *p* ∼10^−17^). See Figures S3E and S3F for the independent analysis of strength and latency.

The normalized firing rate and response latency for all 159 response-eliciting stimuli are shown in Figure 2A. First, the responses to text and sound stimuli exhibit larger latencies than those to pictures. Moreover, the responses to the pictures have been sorted by the latency of the 47 response-eliciting identities, and although they cover a wide 250-ms latency range for the whole dataset, it can be seen that the responses within a given identity are clustered together.

To further understand the response characteristics of these neurons, we first analyzed the degree of visual invariance observed in the single unit responses (Figure 2B). For each response-eliciting identity, we compared the spike count associated with the three pictures against the one of three randomly selected pictures from the other identities (STAR Methods). We found that 36 out of 47 identities (77%) showed significant visual invariance, indicating a tendency to respond to different pictures from a particular identity, which is consistent with previously reported results in the amygdala and hippocampus [20]. For simplicity, we named this invariance, although it does not imply that the responses to the different pictures are indistinguishable, as described below (see Discussion). We also observed that if there was a response to at least one picture from a given identity, the probability of responding to another picture from the same identity was 0.78.

For visual stimuli, we did not observe differences between the responses in the hippocampus and amygdala (Z test, p > 0.64). When text and sound stimuli were considered, 38% and 45% of the identities showed significant responses, respectively, with larger proportions in hippocampus compared to the amygdala. Moreover, 28% of the identities showed visual invariance and significant text and sound responses (two such identities are shown in Figure 1), and when these results were segregated between hippocampus and amygdala, we found that this proportion was significantly larger in the hippocampus (Z test, p < 0.05). All of these results are also consistent with previously reported results [20].

### Neural Responses to Different Stimuli from the Same Identity

Next, we quantified the normalized strength of the activity of every responsive unit to both response- and non-response-eliciting stimuli. As it can be seen in Figure 2C, a neuron showed zero strength in response to most of the non-response-eliciting stimuli, while it responded similarly and with its maximum strength to most of the response-eliciting stimuli. This result points toward a nearly binary code, where neurons mainly respond to the stimuli with maximum (minimum) strength.

Following the idea introduced in [26], we studied whether the neurons showed neural unitization in their responses to a given identity, i.e., if individual neurons exhibited no differences in response strength or latency to the different pictures associated to a given identity. Figure 2D shows the response strength (baseline subtracted) and latency for each triplet of pictures on 25 out of the 47 response-eliciting identities (for space reasons, the remaining 22 are shown in Figures S3A and S3B). Overall, responses show a wide range of strengths and latencies (the full strength and latency distributions can be seen in Figures S3C and S3D), but most identities exhibit responses that appear close to each other (i.e., with similar strength and latency).

To further quantify the similarity between the responses within each identity, we computed the average joint distance between the three pictures of a given identity (normalizing strength and latency between 0 and 1) and compared them with the ones of randomly chosen triplets (i.e., randomly selecting three pictures out of the 141 pictures arising from the 47 response-eliciting identities). Figure 2E shows the distribution of joint distances for the response-eliciting identities and for the random triplets, which were significantly different (rank-sum test, p ∼10^−17^). In Figures S3E and S3F, we repeated the same analysis but separately for strength and latency, finding again significant differences when comparing the response-eliciting identities and the random triplets (rank-sum test, p ∼10^−7^ and p ∼10^−17^, respectively).

### Comparison of Responses to Stimuli from the Same Identity

Using the same methodology as in [26], we analyzed differences in the strength and latency for pairs of stimuli using a permutation test (i.e., shuffling the label of the stimulus shown on each trial). First, we started with the pairs generated from all the stimuli associated to the 47 response-eliciting identities, regardless of whether or not the individual stimuli passed the response criterion. When we applied the permutation test to all 141 pairs of pictures, we found 25% and 10% of them exhibited significant differences in strength and latency, respectively. This is consistent with the results depicted in Figures 2D and 2E that show the similarity of the neural responses across different pictures from the same identity. When considering pairs of stimuli formed by one responsive picture and the written name of the same identity, 57% and 50% of them exhibited significant differences in strength and latency, respectively; these values are significantly larger than those from the pairs of pictures (Z test, p ∼10^−8^ and p ∼10^−13^, respectively). Similarly, when considering pairs with one responsive picture and the spoken name of the same identity, 50% and 49% of them exhibited significant differences in strength and latency, respectively; these values are again significantly larger than those from the pairs of pictures (Z test, p ∼10^−6^ and p ∼10^−12^, respectively).

**Figure 3:**
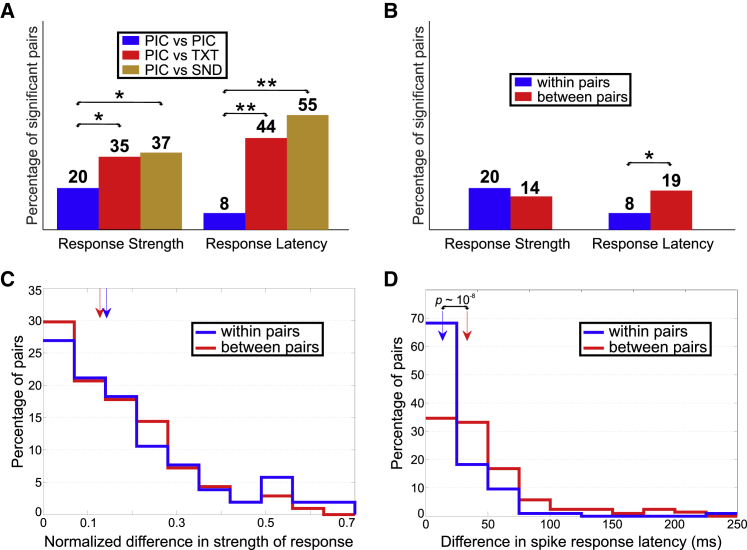
Comparison of Responses within and between Identities (A) Percentage of pairs exhibiting significant differences in strength (left) and latency (right) according to a permutation test for the response-eliciting pairs “within identity.” The pairs could be formed by two responsive pictures (PIC versus PIC), one responsive picture and the written name from the same identity (PIC versus TXT), or one responsive picture and the spoken name from the same identity (PIC versus SND). The percentage was significantly smaller for the PIC versus PIC case when compared with the others. ^∗^p < 0.05; ^∗∗^p < 10^−6^. An alternative analysis was performed using a decoding approach. Figure S4 shows an example of a decoder being unable to significantly distinguish among the stimuli within a given identity. (B) Proportion of pairs exhibiting significant differences in strength (left) and latency (right) according to a permutation test for the response-eliciting pairs “within identity” (from the current dataset) and “between identities” (from the dataset reported in [26], see STAR Methods). These proportions showed no significant differences across the datasets for strength (Z test, p = 0.15), but the proportion for the within identity dataset was significantly smaller than for the between identities dataset (Z test, p = 7.3 × 10^−3^). (C) Distributions of the normalized difference in strength of response for each dataset. Vertical arrows denote the median of the corresponding distributions. There was no significant difference between them (rank-sum test, p = 0.12). (D) Same as in (C) but for differences in latency. We observed that the latency difference was significantly smaller for within identity pairs than for between identities pairs (rank-sum test, *p* ∼10^−8^).

Next, we run the same tests but only for response-eliciting pairs, i.e., where both stimuli in the pair satisfied the response criterion. When the surrogate test was applied to the response-eliciting pairs of pictures from the same identity, we found that only 20% and 8% of them exhibited significant differences in strength and latency, respectively (Figure 3A). The fact that these numbers are similar to the ones obtained for all of the pairs from the response-eliciting identities emphasizes again that most of them were indeed formed by response-eliciting stimuli.

When considering pairs of stimuli formed by one responsive picture and the written name of the same identity, 35% and 44% of them exhibited significant differences in strength and latency, respectively (Figure 3A); these values are significantly larger than those from the pairs of pictures (Z test, p = 0.039 and p ∼10^−7^, respectively). In line with these results, when considering pairs with one responsive picture and the spoken name of the same identity, 37% and 55% of them exhibited significant differences in strength and latency, respectively (Figure 3A); these values are again significantly larger than those from the pairs of pictures (Z test, p = 0.017 and p ∼10^−10^, respectively). The proportion of pairs with significant differences in latency is higher than the one obtained previously for all the pairs because the responses to the spoken name have much larger latencies than those of non-responsive stimuli.

### Predicting Stimuli Based on Single-Trial Spike Count in the Response Period

We further evaluated the proportion of neurons showing differences in strength using a decoding approach. First, we trained a naive Bayesian decoder choosing randomly one response-eliciting stimulus and one stimulus from a non-response eliciting identity, and then used all the other stimuli to test whether the decoder could correctly distinguish between responsive and non-responsive identities. We found that 33 out of 35 neurons (94%) exhibited significant decoding performance (in the other two cases, performance was close to significant with p values of 0.06 and 0.07, respectively).

Then, we used a decoder to see if each picture from the response-eliciting identities could be predicted based on the single-trial response strength (STAR Methods). Figure S4 shows the confusion matrix for the three pictures of “Charlotte Caniggia” that elicited responses in a left hippocampus neuron. As the decoding performance (30%) was not significantly different from chance (p = 0.72), the three pictures were indistinguishable from each other. Across all of the responsive units, it was only possible to predict (above chance) the stimuli to which the neurons responded in 7 out of 35 units (20%), which is consistent with the 20% of pairs with significant differences observed with the surrogate tests described above.

To further emphasize the nearly binary code for each identity, we repeated the decoding analysis after binarizing the responses using a threshold on the strength (STAR Methods), finding a similar percentage of neurons with significant decoding performance (8 out of 35, 23%).

### Responses within and between Identities

We compared the results from this experiment, where pairs of response-eliciting pictures from the same identity were constructed for each responsive unit, with the ones from another dataset reported in [26], where we analyzed pairs of response-eliciting pictures for each responsive unit that came from different identities (STAR Methods).

Figure 3B shows that when permutation tests were applied to the response-eliciting pairs from the dataset reported in [26] (“Dataset 2”; between identity pairs), 14% of the pairs showed significant differences in strength, a percentage not significantly different to the 20% observed in the current dataset (“Dataset 1”; within identity pairs; Z test, p = 0.15). In addition, we computed the distributions of the normalized difference in strength of response for each dataset (Figure 3C) and found no significant difference between them (rank-sum test, p = 0.12). However, the results were different when we performed the analyses on the spike response latency. Using the permutation tests, we found that 19% of the response-eliciting pairs in Dataset 2 showed significant differences in latency (Figure 3B), which was a significantly larger proportion than the 9% observed in Dataset 1 (Z test, p = 0.007). Moreover, when comparing the distributions of the difference in spike response latency (Figure 3D), we observed that the latency difference was significantly smaller for “within identity pairs” than for “between identity pairs” (rank-sum test, *p* ∼10^−8^). This could be due to the presence of a larger set of neurons responding to pictures of the same identity, compared with those responding to associated pictures, thus homogenizing the latency of the responses in the former case. To rule out that this result could be associated to a difference in the distribution of response latencies across datasets (e.g., the latencies in Dataset 1 being significantly smaller than in Dataset 2), we compared them and found no significant difference between them (rank-sum test, p = 0.41).

## Discussion

Here, we described visually invariant responses in the human MTL, in the sense that the responses to different pictures were mostly indistinguishable from each other based on the response strength and latency. This is in contrast to the typical finding of graded responses in the neocortex [1, 2]. IT neurons respond to complex visual features, like specific objects, and show graded responses to object rotations but, at the same time, some degree of invariance to simple image manipulations, such as size and position [4, 5, 6, 7, 8, 9]. However, in this context, invariance is taken as a preserved selectivity across large variations of these parameters (i.e., the neuron continues to have a stronger response to a particular item) but without necessarily implying the same neuronal response in all conditions. In fact, in these cases, it is typical to observe a clear tuning of the neurons’ responses [1, 2, 4, 16, 17], and differences in the responses can be used to distinguish the response-eliciting stimuli [8, 9, 16, 17, 18]. A large number of studies has shown responses of IT neurons to faces [9] but also with a graded coding [7], particularly when shown with different orientations [10, 11] or levels of morphing [12], among other manipulations.

In previous works [20, 21], we have shown that MTL neurons tend to fire to pictures of specific persons (and to the persons’ written and spoken names), but because of the relatively low number of trials used in these studies (six trials per stimuli), we could not statistically compare the responses to the different pictures (and names) of the same persons. To address this issue, in this study, we have used 30 presentations per stimulus and, in contrast to the findings in the neocortex studies described above, we have shown that MTL responses were nearly binary, meaning that response-eliciting stimuli triggered the same activations while other stimuli elicited activations at baseline levels—i.e., without showing a graded coding. The finding of an indistinguishable response strength cannot be attributed to a choice of a relatively large threshold for defining responses. In fact, responses of different neurons covered a wide range of firing above baseline (see Figures 2D and S3C), and furthermore, we reduced the response threshold criterion (from 5 SD to 4 SD) to avoid artificially separating responses that were close to this value; however, we kept a criterion of 5 SD for initially defining responsive identities to avoid too many false positives (see Figure S1). Moreover, a single-trial decoding approach, which does not rely on any responsiveness criterion, gave very similar results. However, different results were seen when considering the responses to the written and spoken names. In this case, the responses to the names tended to be different than the ones to the pictures, likely because of very different cortical inputs.

MTL neurons do not act in isolation but are rather part of cell assemblies representing specific concepts [22]. These assemblies are very sparse, eliciting the firing of about 0.2% of the neurons to a given familiar concept (e.g., a particular person) [27]. About 70%–80% of these neurons fired indistinguishably to different pictures of this particular person. Therefore, it is not possible from the firing of the individual neurons to distinguish between different persons eliciting the neurons’ responses, and such a distinction is determined by the activation of different neural assemblies. Given the graded responses in cortex, we can then postulate that different pictures of a particular identity trigger different cortical representations that initially ignite different subsets of an MTL cell assembly but rapidly activate most of it through pattern completion.

**Figure 4:**
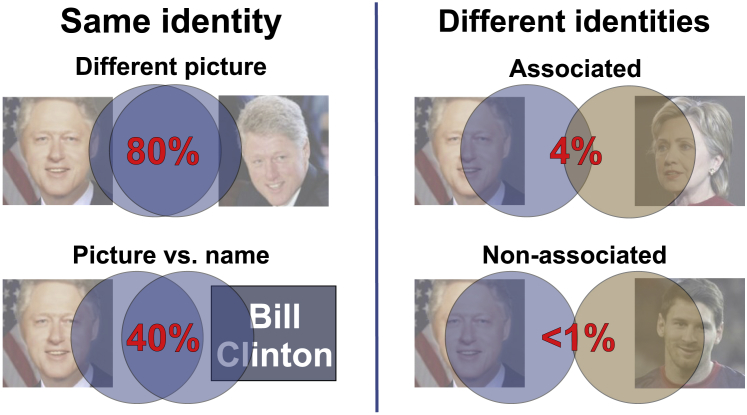
Schematic Summarizing Some of the Results When a picture from Bill Clinton is shown, a cell assembly of MTL neurons will fire in response to the stimulus. When another picture from Bill Clinton is presented, the set of MTL neurons firing has a large overlap (∼80%) with the previous assembly. When the written name is presented, the overlap of activated neurons is much lower (∼40%), as the very different sensory information carried by the name leads to very distinct cortical inputs to the MTL. In turn, the results from [28] showed that when the picture of Hillary Clinton is presented, a highly associated concept to Bill Clinton, a small overlap of ∼4% will be activated by both stimuli. Still, this small proportion is much larger than the < 1% observed for non-associated pictures (e.g., Bill Clinton and Lionel Messi), providing a substrate for encoding a meaningful association.

In a previous study, we showed that MTL neurons tend to respond to associated identities [28]. Moreover, we estimated that given a response to a first identity, the probability that a MTL neuron will fire to a second highly associated identity (e.g., Bill Clinton and Hillary Clinton in Figure 4) is about 4% [28]. Furthermore, the responses to associated identities have similar strength and latency—meaning that at the single neuron level, it is not possible to distinguish two identities eliciting the neuron’s responses [26]. The probability of the neuron responding to non-associated identities is much lower (< 1%; e.g., Bill Clinton and Lionel Messi in Figure 4) but can be rapidly raised if the identities become associated [29]. Putting together this evidence, we can postulate that the largely overlapping assembly activation in the MTL by different pictures of the same identity (∼80%; Figure 4) leads to unitization at the behavioral level [30]—i.e., the fact that two pictures of the same concept convey the same meaning—whereas different but associated identities give a much lower but still significant overlap (∼4%), which is low enough to distinguish the identities from each other, yet, at the same time, large enough to encode meaningful associations that may eventually lead to temporary coactivations. Responses to the written or spoken names are in between (∼40%; Figure 4), which allows the ability to identify that two stimuli refer to the same identity but still distinguishing between the picture and the written name.

Studies in animals and patients have demonstrated the key role of the MTL in declarative memory [23, 25, 31, 32, 33]. Within this framework, it has been argued that the high-level invariant responses by “concept cells” in the human MTL represent the meaning of the stimulus for declarative, and particularly episodic, memory functions [21, 22]. Episodic memories rely on the encoding of associations between concepts [22, 24, 34, 35]. Contrasting with the graded responses in neocortex [4, 5, 6, 9, 10, 11, 13, 14, 15, 18], which may be useful for recognition in order to detect changes in the environment, we can postulate that the degree of visual invariance shown by MTL neurons leads to a high degree of conceptualization and abstraction that is the basis of our memory functioning, because we tend to remember concepts and forget irrelevant details [22]. Differences in the responses between different types of stimuli (i.e., pictures versus written and spoken names) may allow the type of information constituting a particular memory—i.e., whether we saw a particular event, heard, or read about it—to be distinguishable at the MTL-neuron level.

The high degree of (particularly visual and, to a lesser extent, also multimodal) invariance found in this study dramatically reduces the amount of information that needs to be stored in memory, at the cost of discarding perceptual features. Moreover, the invariant coding by concept cells gives a representation that is critical in order to understand memory functioning and its capacity, offering several advantages, such as efficiency, robustness, flexibility, and ease of readout [36, 37]. We argue that such code is ideal for the fast and efficient formation [29] and long-term coding [28] of episodic memories, which are complemented by sensory feature information stored in neocortex.

## STAR★Methods

### Key Resources Table

**Table:** 

REAGENT or RESOURCE	SOURCE	IDENTIFIER
**Software and Algorithms**		

MATLAB	MathWorks	https://www.mathworks.com
Wave_clus	[38] [39]	https://github.com/csn-le/wave_clus
Custom-built MATLAB code	This paper	https://www2.le.ac.uk/centres/csn/software
Psychtoolbox version 3	[40]	http://psychtoolbox.org/
Text Aloud v3.0	NextUp Technologies	https://nextup.com

**Deposited Data**		

Spiking activity for the responses in the current dataset	This paper	10.25392/leicester.data.11527983

### Lead Contact and Material Availability

Further information and requests for resources should be directed to the Lead Contact, Rodrigo Quian Quiroga (rqqg1@le.ac.uk). This study did not generate new unique reagents.

### Experimental Model and Subject Details

We report results from 16 experimental sessions in 6 patients with drug resistant epilepsy that were candidates for surgery (all right-handed, three males, 19-49 years old). Patients were implanted with chronic depth electrodes at “Hospital El Cruce” in Buenos Aires, Argentina. They were monitored 24/7, for seven to ten days, to determine the epileptogenic region for possible surgical resection [41]. A written informed consent was signed by each patient to participate in this study. All the experimental procedures were carried out in accordance with the Declaration of Helsinki and approved by “Hospital El Cruce” Medical Institutional Review Board.

### Method Details

#### Electrophysiological Recordings

Each electrode probe had a total of nine microwires at its end, eight active recording channels and one (low impedance) reference (AD-TECH Medical Instrument Corporation, Wisconsin, USA). Electrode locations in hippocampus (11 probes) and amygdala (8 probes) were based exclusively on clinical criteria and were verified by CT co-registered to preoperative MRI. The signals were recorded using a 128-channel Cervello® Elite EEG System (Blackrock Microsystems, UT, USA), filtered between 0.3 and 7,500 Hz, and sampled at 30,000 Hz.

#### Experimental Paradigms

As in previous works [21, 41], a simple visual task was used to identify responsive stimuli. A standard laptop running the Psychophysics Toolbox (www.psychtoolbox.org/ [40]) under MATLAB (https://www.mathworks.com) was used for stimulus presentation. The subject was sitting facing the laptop in which a set of about 100 stimuli were presented, 6 times each in pseudorandom order using a block design (i.e., if N stimuli are used in the session, all stimuli will be shown once, in random order, after the first block of N trials, twice after the first 2^∗^N trials, etc.). Each trial started with a fixation cross on the screen for 500 ms, followed by a picture displayed for 1,000 ms. Then, the screen went black and the patient had to press a key to respond whether or not there was a person in the picture. The inter-trial interval varied randomly between 600 and 800 ms. These ‘screening sessions’ typically lasted about half an h. The set of pictures used included items familiar to the patient, such as images of celebrities, landmarks, animals, and the patient’s relatives and friends, as these tend to trigger MTL neuron responses [42].

Once it had been identified which picture/s triggered the firing of which neuron, between 3 and 5 of them were selected for a follow-up session. This two-step approach has been successfully used in the past [26], where we explicitly showed that this picture selection did not introduce a bias toward high firing rate responses (i.e., we did not miss intermediate responses). In the experiment presented here, a total of 5 stimuli were presented for each identity: 3 different pictures (one of them being the responsive picture from the initial screening session), the name written on the screen, and the name spoken by a computer-synthesized voice (TextAloud v3.0; https://nextup.com). Each of these stimuli were shown 30 times in pseudorandom order. The data reported here come from these follow-up sessions. It should be emphasized that since we want to compare the responses to different stimuli from a given identity, a selection of a subset of responsive stimuli from the screening session does not introduce a bias in our results, as we did not know in advance how the neuron will respond to the new 4 stimuli per identity presented in the follow-up session. Moreover, by excluding the pictures already presented during the screening sessions, we found that if there was a response to at least one of the novel pictures from a given identity, the probability of responding to the other picture from the same identity was 0.75. This result with only the novel pictures is similar to the 0.78 observed for all the pictures.

### Quantification and Statistical Analysis

#### Single neuron response criterion

The collected data were processed offline, and the high-frequency activity (above 300 Hz) was extracted to identify the spikes of the recorded neurons. Spike detection and sorting was done with Wave_clus [38, 39]. In order to assess whether a particular unit was responsive to a certain stimulus, the following response criterion was implemented [26]: (i) the instantaneous firing rate had to cross over a threshold for at least 75 ms (with the upward crossing defined as *t*_THR_), with short periods of less than 20 ms going below threshold being disregarded. The instantaneous firing rate was calculated by convolving the spike train with a Gaussian kernel with σ = 10 ms (truncated at 1% amplitude). The threshold was set to the mean plus 4 standard deviations, computed across all stimuli between 900 and 100 ms before stimulus onset (with a minimum at 5 Hz, for neurons with low baseline firing); (ii) the median number of spikes (across trials) in a 500-ms window from *t*_THR_ was at least 2, and larger than the mean plus 5 standard deviations (across all stimuli) of the baseline activity, defined as the median number of spikes (across trials) between 200 and 700 ms before stimulus onset (i.e., Z-score > 5); (iii) the *p-value* of a one-sided paired sign test between the spike count on each trial for the post stimulus (500-ms window from *t*_THR_) and baseline (200 and 700 ms before stimulus onset, for the particular picture) was less than 0.01. In addition, *t*_THR_ was defined as the spike response latency onset.

From the 450 units recorded in the follow-up sessions, the response criterion led to a set of 35 responsive units (16 from the amygdala, 19 from the hippocampus). All the observed responses came from areas not associated with the epileptogenic zone defined *a posteriori* by the neurologists. Based on a previously used criterion [43], we classified units as single- or multi-units based on: (i) the spike shape and the variance of the cluster; (ii) the ratio between the peak value of the mean waveform and the standard deviation at their first sample (larger than 5, for single-units); (iii) the ISI distribution of each cluster; and (iv) the presence of a refractory period for the single units, i.e., < 1% spikes with an ISI smaller than 3 ms. This way, we identified 4 out of the 35 units as multiunits, with the remaining 31 classified as single units.

The response criterion led to 134 responses, corresponding to 47 identities, in 35 units. However, we observed that some of the 101 stimuli that did not pass the response criterion, from the total of 235 associated to the 47 response-eliciting identities, could actually be described as relatively good responses based on visual inspection. In fact, by decreasing the threshold on the Z-score from 5 to 4 (in the second condition of the response criterion), 25 out of the 101 stimuli passed the criterion, leading to a total of 159 responses. Figure S1 shows three examples of these responses. Relaxing the criterion over the whole set of recordings led to 12 additional identities with a single “responsive stimulus” that were actually very poor responses based on visual inspection, which is why we used the dual threshold criterion to first determine responsive identities and later responsive stimuli.

#### Visual Invariance

Visual invariance was quantified for each response-eliciting identity, using a rank-sum test to compare the spike count over a 700-ms window from the spike response onset for the 3 pictures of a given identity, against a set of 3 randomly selected pictures, one from each of the other identities presented in the same follow-up session. When a stimulus did not elicit a response, the window onset was taken as the average spike response onset across the response-eliciting stimuli (excluding the sound), with an extra 100ms added in the case of sound stimuli as they have typically longer latencies. This analysis was repeated 1000 times, leading to a thousand *p-value*s, from which we took their median as the definitive *p-value* for the concept under analysis. An identity was considered visually invariant when the *p-value* was less than 0.01.

#### Quantification of single neuron response strength

The strength of spike activity was defined as the median number of spikes (across trials) fired by a unit in a given time window, normalized by the window length (Figures 2 and 3). During the baseline period, it was computed between 1,000 and 300 ms before stimulus onset; whereas the strength of individual responses was calculated in a 700-ms window starting at the spike response onset.

To quantify the normalized strength shown in Figure 2C, first we measured, for each responsive unit, the median number of spikes across trials between 100 and 800 ms after stimulus onset for every stimulus. Then we corrected (subtracted) the activity by the mean baseline (across all stimuli) and normalized it by the maximum across all stimuli. When a unit showed activity below baseline for a certain stimulus, it was assigned a zero strength.

#### Quantification of differences in response strength

Differences in response strength were assessed with a permutation test. Given a pair of responses from the same unit, we compared their absolute difference in strength of response ΔS_r_ to a distribution of 1000 surrogate values, created by randomly permuting the trial labels for the two stimuli. Specifically, for each re-arrangement of the labels, we obtained two surrogate responses and calculated their absolute difference in strength of response (ΔS_*i*_). The ranking of the real value (ΔS_r_) among the population of surrogate values, gave the *p-value* for the null hypothesis that the two responses had the same strength. We have previously shown that the 30 trials presented for each stimulus are enough to get a reliable estimate of the strength difference [26].

#### Study of single neuron response latencies

We used a surrogate test implementation similar to the one used for response strength to compare the latencies associated to a pair of stimuli. For this, we calculated the difference in latency of response (ΔL_r_) for each response pair and, as above, for each comparison the *p-value* was obtained by comparing the real test statistic value with 1000 surrogate values. We have previously shown that the 30 trials presented for each stimulus are enough to get a reliable estimate of the latency difference [26]. When this test was applied to stimuli that are not necessarily responsive, if a latency could not be defined as explained above in the response criterion, the first threshold crossing in the firing rate was taken as the latency.

#### Decoding analysis

A naive Bayesian decoder with leave-one-out cross-validation was run on each responsive unit to test whether the identity of the individual stimuli associated to the responsive identities could be predicted based on the single trial spike count in the response period (Figure S4). The decoding performance was estimated as percentage of trials correctly predicted, and its statistical significance was assessed in comparison to the performances obtained on a population of 1000 surrogates created by randomly shuffling the trial labels. In addition, we repeated the analysis after binarizing the responses. To do this we defined a threshold for each unit. First, we computed the mean strength (*mstr*) across trials for each stimulus. Then, we defined the threshold as the average between the maximum and minimum values of *mstr* across all stimuli.

#### Comparison with responses to different identities

In Figure 4, we compared the results from the current experiment (Dataset 1) with those reported in [26] (Dataset 2), where we analyzed the responses of individual units to different pictures of associated identities. Dataset 2 was obtained by performing follow-up sessions in which a subset of about 15 stimuli (mean ± SD, 13.9 ± 4.5) from the screening session (including all those that elicited a response) were used, but each of these images was shown 25–35 times in pseudorandom order. We found 37 multi-responsive units (i.e., units exhibiting responses to more than one picture), which responded to an average of 3.3 pictures (SD: 1.94). By identifying the response-eliciting pairs of stimuli in each of these units, we defined a total of 208 pairs. The response criterion employed in Dataset 2 used Z-score > 5. For comparison purposes, we repeated the analyses in Figure 4 using Z-score > 5 in Dataset 1, leading to the same qualitative results.

### Data and Code Availability

The response dataset associated to this manuscript can be downloaded from 10.25392/leicester.data.11527983. The main (spike sorting) codes used to process the data can be downloaded from https://www2.le.ac.uk/centres/csn/software.

## Supplemental Information

Document S1. Figures S1–S4

Document S2. Article plus Supplemental Information
