# Supplementary material for: Single Neuron Coding of Identity in the Human Hippocampal Formation
Source: Curr Biol. 2020 Mar 23;30(6):1152–1159.e3. doi: 10.1016/j.cub.2020.01.035 (PMC7103760; doi:10.1016/j.cub.2020.01.035)
Supplement: Document S1. Figures S1–S4 [file mmc1.pdf]

Current Biology, Volume *30*

Supplemental Information

Single Neuron Coding of Identity in the Human Hippocampal Formation

Hernan G. Rey, Belen Gori, Fernando J. Chaure, Santiago Collavini, Alejandro O. Blenkmann, Pablo Seoane, Eduardo Seoane, Silvia Kochen, and Rodrigo Quian Quiroga

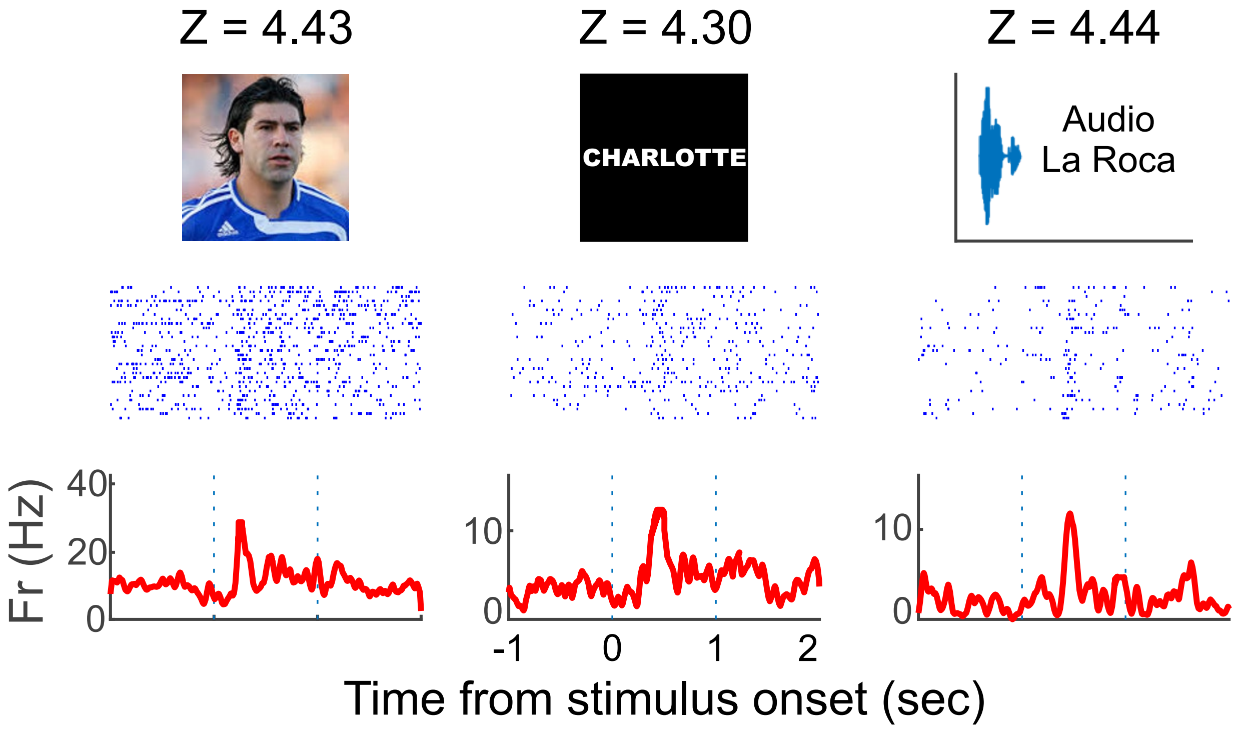

Figure S1. Response criterion. Related to Figures 1 and 2A. Exemplary new responses of picture, text, and sound stimuli when the response criterion was relaxed from Z-score > 5 to Z-score > 4. Same conventions as in Figure 1.

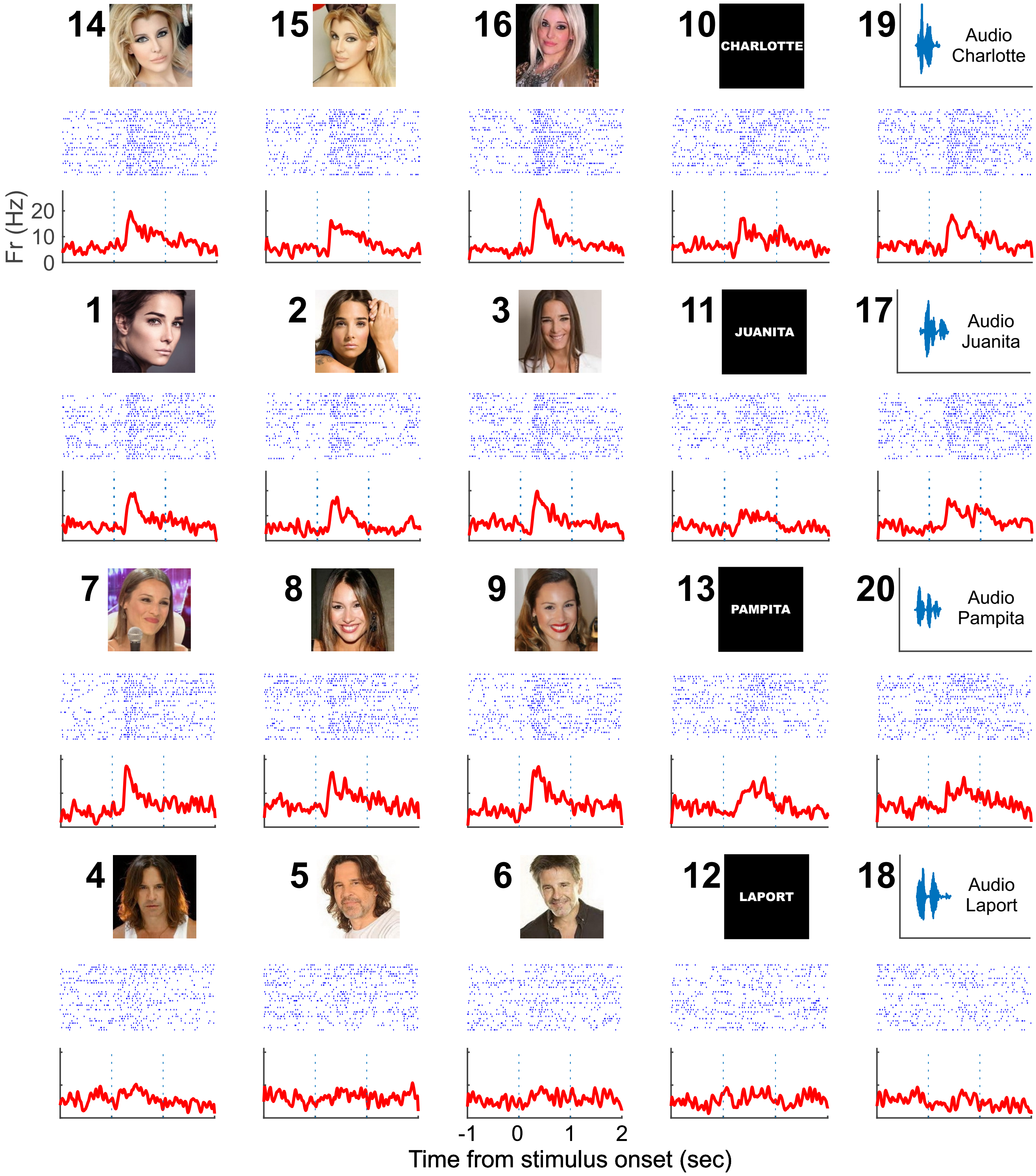

Figure S2. Example of an invariant multi-responsive unit. Related to Figure 2A. Responses of a unit in the left hippocampus. Same conventions as in Figure 1. The unit responded to the stimuli associated to three different celebrities ("Charlotte Caniggia", "Pampita", and "Juanita Viale"), but not to a fourth one ("Osvaldo Laport"). All these celebrities took part on the TV show ("Showmatch").

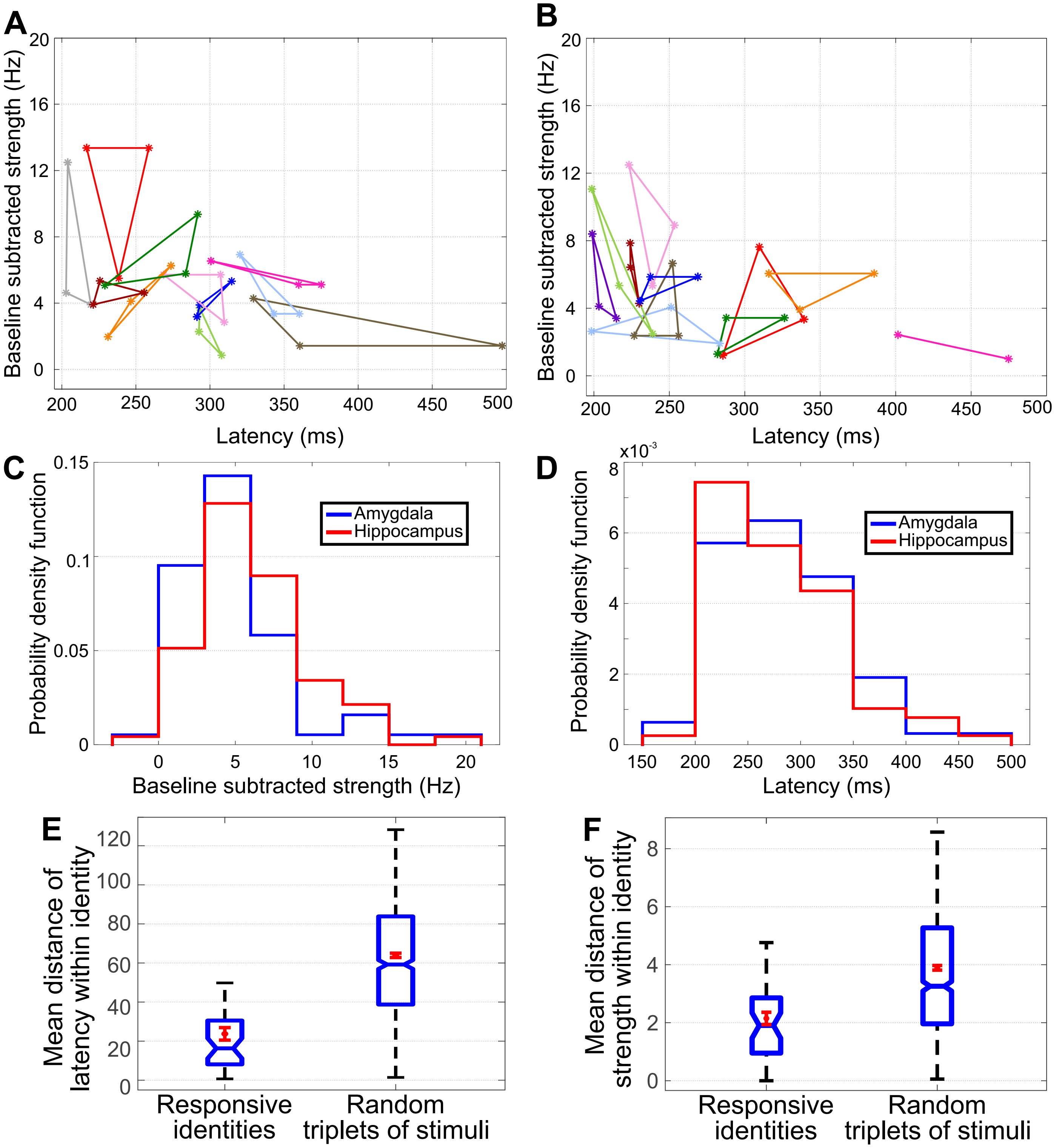

Figure S3. Similarity of strength and latency of responses within identity. Related to Figures 2D and E. (A) and (B) Joint representation of strength and latency for 22 exemplary response-eliciting concepts (the remaining 25 are shown in Figure 2D). Same conventions as in Figure 2D. The identities are split in panels A and B for clarity. (C) and (D) Strength (baseline normalized) and latency distributions of responses to all the pictures associated with the 47 response-eliciting identities. No differences were seen between amygdala and hippocampus (two-sampled t-test, $p>0.05$). (E) Same analysis as in Figure 2E but with the average distance computed marginally for the latency of response. There were significant differences when comparing the response-eliciting concepts with the random triplets (rank-sum test, $p \sim 10^{-17}$). (F) Same as in (E) but with the average distance computed marginally for the strength of response. There were significant differences when comparing the response-eliciting concepts with the random triplets (rank-sum test, $p \sim 10^{-7}$).

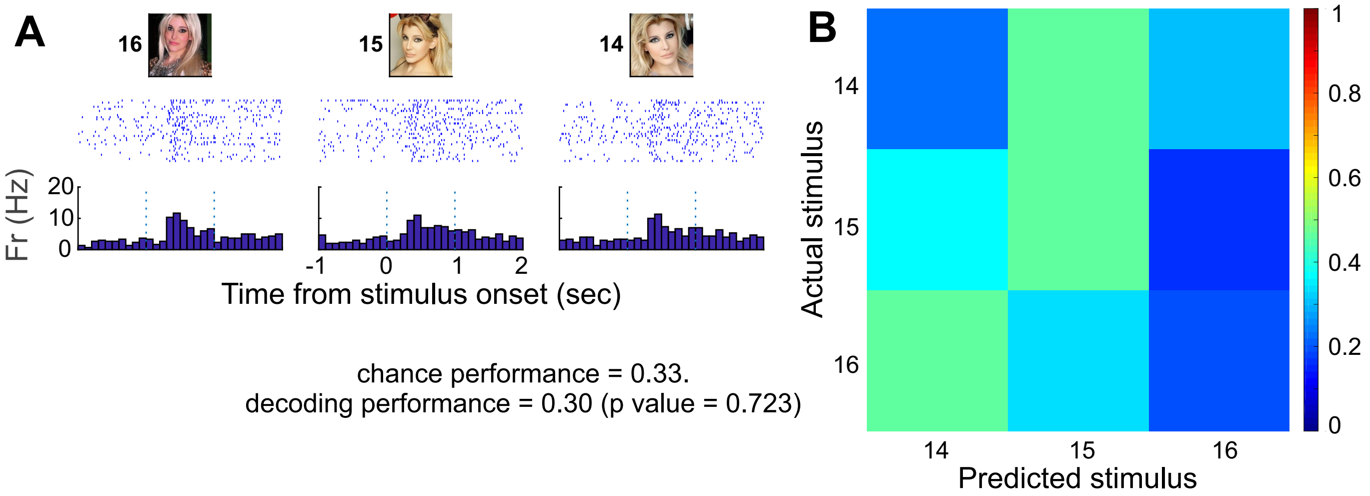

Figure S4. Example of decoding performance. Related to Figure 3A. (A) Raster plots for 3 pictures of Charlotte Caniggia from a left hippocampus neuron. (B) Confusion matrix showing the decoding performance that was not different from chance, meaning that the stimuli could not be distinguished based on their strength.
